# Case report: Local bleomycin injection: A possible treatment option for primitive myxoid mesenchymal tumor of infancy

**DOI:** 10.3389/fped.2022.993450

**Published:** 2022-10-11

**Authors:** Binbin Yang, Qingjiang Chen, Yueling Zhu, Jianbing Wang, Ao Dong, Yi Chen, Xue He, Weizhong Gu, Zhigang Gao, Yunzhong Qian

**Affiliations:** ^1^Department of General Surgery, National Clinical Research Center for Child Health, National Children's Regional Medical Center, The Children's Hospital, Zhejiang University School of Medicine, Hangzhou, China; ^2^Department of Traditional Chinese Medicine, National Clinical Research Center for Child Health, National Children's Regional Medical Center, The Children's Hospital, Zhejiang University School of Medicine, Hangzhou, China; ^3^Department of Epidemiology and Biostatistics, National Clinical Research Center for Child Health, National Children's Regional Medical Center, The Children's Hospital, Zhejiang University School of Medicine, Hangzhou, China; ^4^Department of Clinical Laboratory, National Clinical Research Center for Child Health, National Children's Regional Medical Center, The Children's Hospital, Zhejiang University School of Medicine, Hangzhou, China; ^5^Department of Nephrology, National Clinical Research Center for Child Health, National Children's Regional Medical Center, The Children's Hospital, Zhejiang University School of Medicine, Hangzhou, China; ^6^Department of Pathology, National Clinical Research Center for Child Health, National Children's Regional Medical Center, The Children's Hospital, Zhejiang University School of Medicine, Hangzhou, China

**Keywords:** primitive myxoid mesenchymal tumor of infancy, bleomycin injection therapy, necrosis response, tumor necrosis, surgical prognosis

## Abstract

In recent years, it has been determined that primitive myxoid mesenchymal tumors of infancy (PMMTI) are solid tumors. To date, very few cases of PMMTI have been reported, and there is no consensus regarding treatment. To provide additional references, it is necessary to collect and report the diagnoses and treatment outcomes of related cases. We report the case of a 38-day-old girl who presented with a 5-cm purple tumor in the right shoulder. Upon hospital admission, the patient received an intratumoral injection of bleomycin after diagnosis of a possible lymphangioma. 10 days after the treatment, the tumor began to develop inflammation and necrosis, resulting in a clear demarcation between the tumor and surrounding tissue. Hence, during the second hospitalization, we performed a successful tumor resection. Postoperatively, the tumor was pathologically diagnosed as PMMTI. 3 months after excision, the patient showed no local recurrence on re-examination. To the best of our knowledge, this is the first report of a PMMTI in which bleomycin, or other similar chemotherapeutic drugs, have been injected into tumors. This result offers novel insights into the treatment of PMMTI. Injection therapy with bleomycin and similar chemotherapeutics may result in specific responses to PMMTI, which may help in developing better surgical conditions or improving outcomes in non-surgical patients.

## Background

Primitive myxoid mesenchymal tumor of infancy (PMMTI) was first reported by Alaggio et al. (1). It is described as a diffuse growth of spindle, polygonal, and round cells in a myxoid background, in which a delicate vascular network can be observed. It has diffuse reactivity to vimentin but no reactivity to smooth muscle actin, desmin, myogenin, and cytokeratin. To date, approximately 30 cases of PMMTI have been reported (2). The disease is most prevalent in the 1st year of life. A previous study identified internal tandem duplication (ITD) of B-cell CLL/lymphoma 6-interacting co-repressor (BCOR) in PMMTI, which is identical to the defining characteristics of kidney clear cell sarcomas (3). The current treatment consensus for PMMTI prioritizes radical surgery (2). It is occasionally reported that patients who cannot have their tumor completely removed show a high likelihood of postoperative recurrence and distant metastasis (2, 4).

In this study, we report the case of a 38-day-old girl with a PMMTI in the right shoulder, who was intratumorally injected with bleomycin. To the best of our knowledge, this is the first report of a PMMTI in which bleomycin, or other similar chemotherapeutic drugs, have been injected into tumors.

## Case presentation

**Figure 1:**
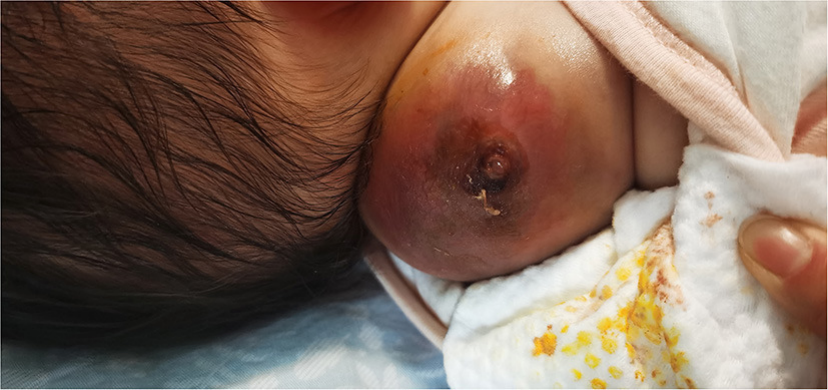
Tumor 10 days after bleomycin injection.

**Figure 2:**
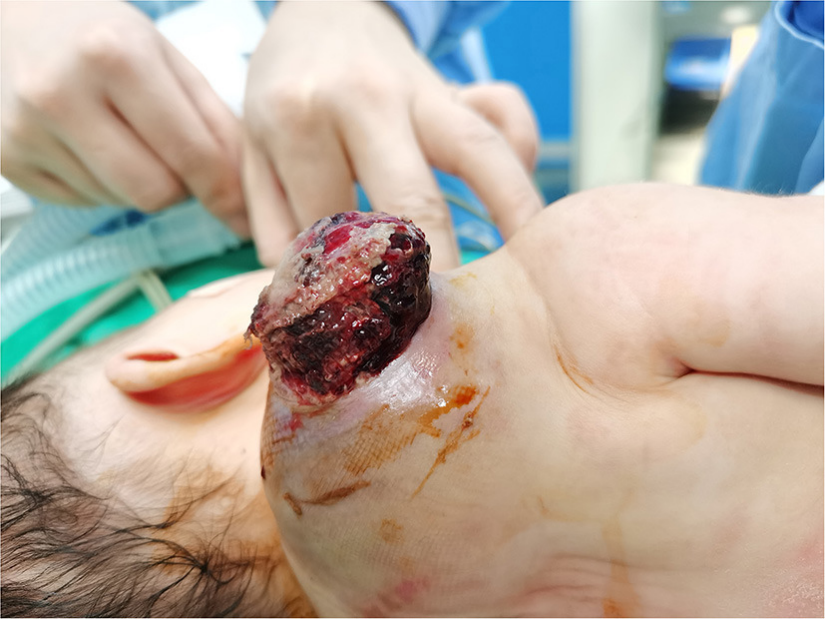
Tumor after 1 week of cefmenoxime treatment.

**Figure 3:**
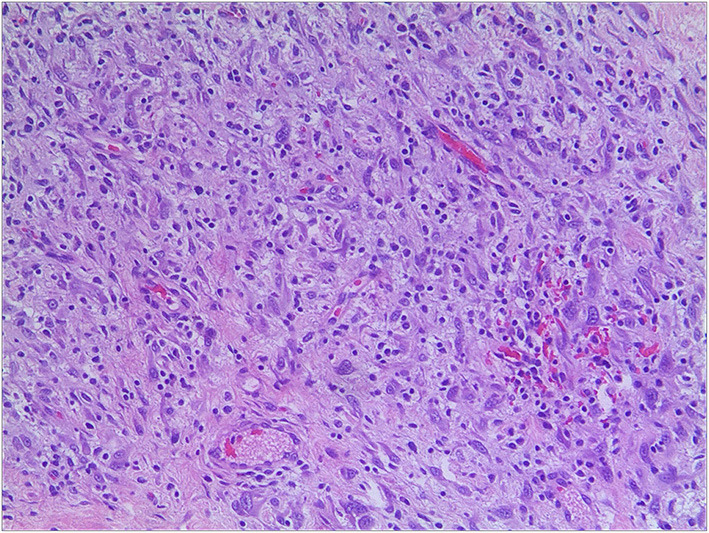
Pathological section of tumor with myxoid background, round and polygonal cells (hematoxylin-eosin, original magnification x200).

**Figure 4:**
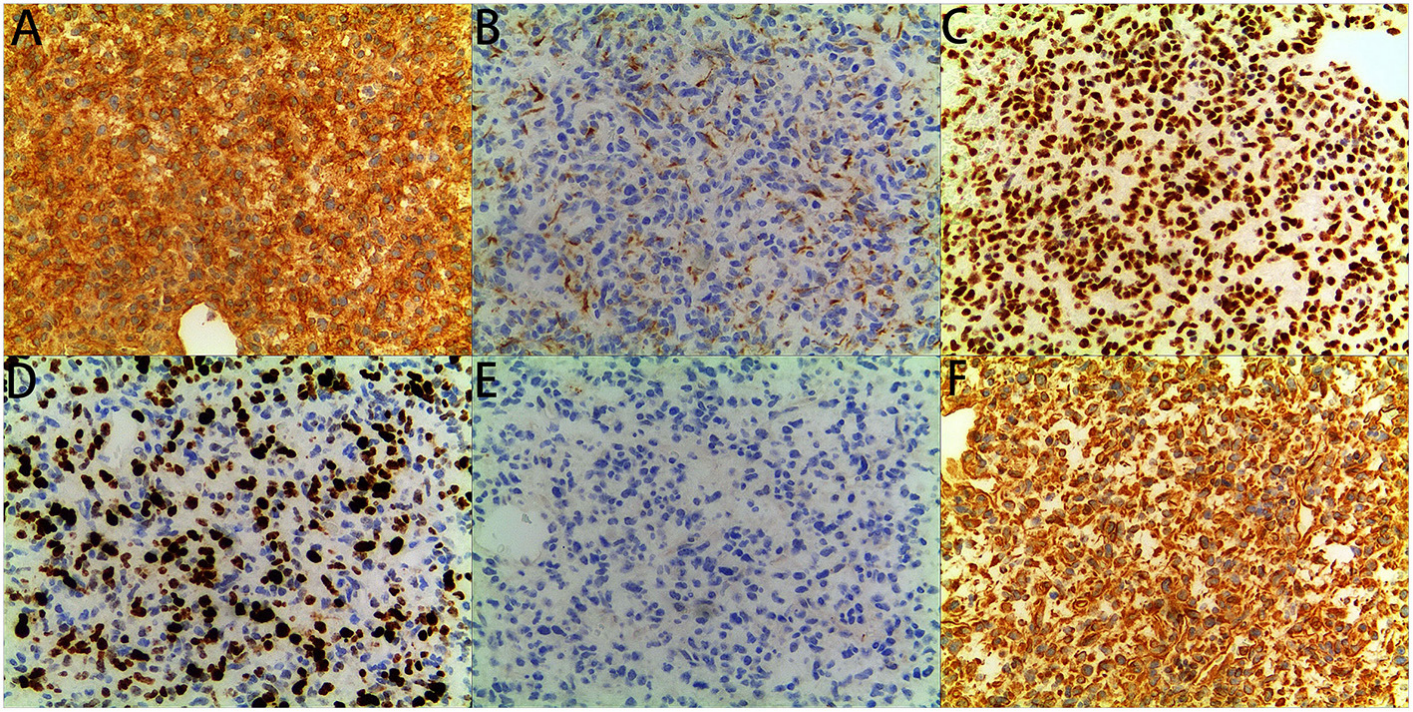
immunohistochemical staining: The tumor showed diffuse immunoreactivity for **(A)** CD99, **(C)** INI1, **(D)** Ki-67, and **(F)** Vimentin, showed Partial immunoreactivity for **(B)** CK (AE1/AE3), showed no immunoreactivity for **(E)** MyoD1 (original magnification x200, for all).

The patient was a 38-day-old girl with a normal vaginal birth at 40 weeks of gestation. Her birth weight was 2,800 g. Her parents were healthy and unrelated. Her mother denied any use of teratogenic drugs, smoking, alcohol consumption, or a history of chronic disease during pregnancy. The patient had a sister who was in good health. Her parents discovered a 5-cm purple tumor on the patient's right shoulder 1 week after receiving the Bacillus Calmette-Guérin (BCG) vaccine; however, there was an absence of pain, swelling, fever, dyspnea, vomiting, and other symptoms. The tumor persisted for 1 week; the texture became slightly more rigid, although its size remained essentially unchanged. The patient was admitted to the local hospital for treatment, and magnetic resonance imaging (MRI) revealed a possible lymphangioma. Subsequently, they visited our hospital's outpatient department where B-ultrasound revealed lymphatic malformation in the right shoulder with bleeding. Routine blood tests, coagulation spectrum, liver and kidney function, preoperative immunity, and necessary preoperative tests, including chest X-ray and electrocardiogram, revealed no obvious abnormalities following admission. On the 2nd day of admission, ~3 mg of bleomycin was administered through multiple injections, and no obvious fluid was removed. Normally, bleomycin would be injected at a dose of 0.3–0.6mg/kg, and the maximum dose should be no more than 10 mg per injection (5, 6). Considering the patient's small weight and large tumor volume, we chose a dose of 0.6 mg/kg. After treatment, the patient did not experience any typical drug reactions, such as fever, vomiting, abdominal pain, or diarrhea, and was discharged the next day after the injection treatment. 10 days after treatment, the tumor became red and swollen, and the surrounding skin began to rupture (Figure 1). Therefore, the patient was readmitted to the hospital. Considering that the patient's tumor may have had a drug reaction to the infection, she was given cefmenoxime 0.15g twice a day for anti-infection; however, there was no discernible improvement, and the necrosis continued to spread (Figure 2). Tumor resection was later performed, and the resected tumor was approximately 6.5 cm × 5.5 cm × 2.5 cm in size. The tumor was pathologically diagnosed as PMMTI (Figure 3). Immunohistochemical detection of the following monoclonal antibodies and oncogenes was assessed: CD3 (-), CD20 (-), CD79a (-), Desmin (partial +), CK (AE1/AE3) (-), CD34 (vascular+), CD31 (+), SMA(vascular+), S-100(-), ALK(-), INI1(present), CD99(+), Bcor(-), MyoD1(-), Myogenin(-), Ki-67(About 30%+), CD117(-), Synaptophysin (-), CD56 (partial +), CD30(-), Ki-67 (about 30%+), and Vimentin(+) (Figure 4). An outpatient follow-up MRI performed 1 month after the patient was discharged from the hospital, revealed no obvious tumor recurrence. During the telephone follow-up ~3 months after surgery, the wound had healed well, and no obvious tumor recurrence was detected.

## Literature review

The diagnosis of PMMTI was proposed by Alaggio et al. (1). They reported six cases of invasive focal tumors characterized by light microscopy as diffuse growth of spindle, polygonal, and round cells in mucus with a myxoid background; delicate vascular networks were visible in many areas. Immunohistochemical analysis revealed a diffuse reaction to vimentin but no reaction to smooth muscle actin, desmin, myogenin, or cytokeratin (1).

Sarcomas in infancy are relatively rare, the most common being embryonal rhabdomyosarcoma, Ewing sarcoma/primitive neuroectodermal tumors, congenital infantile fibrosarcoma, and undifferentiated sarcoma (1). The treatment and prognosis of PMMTI differ from those of these diseases; therefore, a precise diagnosis is essential for the treatment of PMMTI. PMMTI is primarily diagnosed based on pathological histomorphological characteristics and immunohistochemistry. In 2016, Kao et al. discovered an ITD of the *BCOR* gene in undifferentiated round cell sarcoma and PMMTI in infants. Of the seven PMMTI cases reported, six showed BCOR-ITD, which was gradually recognized in later case reports as a diagnostic basis for PMMTI (3).

In all the reported cases, complete resection was recommended as the preferred treatment (2). There are no established treatment guidelines for tumors that cannot be completely resected. PMMTI has been reported to be partially responsive to doxorubicin- and ifosfamide-containing chemotherapy regimens (7, 8), but not to vincristine, actinomycin D, cyclophosphamide (VAC), or VA regimens (4, 8, 9). Of the two patients who received radiotherapy, one had no tumor recurrence at the 12-month follow-up after treatment, whereas the in the other patient, the tumor recurred and transformed into undifferentiated sarcoma (5).

PMMTI usually have a long clinical course; local recurrence after surgery is common, whereas distant metastasis is relatively rare. According to the reported treatment data, 18 patients chose surgical resection as their initial treatment, of which eight showed tumor recurrence after resection, three had metastases, and seven patients had no postoperative recurrence. Two of the seven cases with no residual resection showed local tumor recurrence, seven cases had positive margins, and all cases had tumor recurrence after surgery (2, 10). Considering the current treatment outcomes of surgical resection cases, we believe that complete resection and the establishment of negative margins are key to effective treatment.

## Discussion and conclusion

At the initial visit for the PMMTI described in this case report, both B-ultrasound and MRI revealed lymphangioma. In addition, because the tumor was large and its border was unclear, surgical resection would have resulted in a large wound and potentially compromised limb function. Therefore, we chose bleomycin for the injection treatment, and excision was performed after the tumor was partially reduced. In previous reports, many PMMTI have been identified by the existence of cystic or vascular structures (1), but there is no precedent for injection therapy with chemotherapy drugs, such as bleomycin. Combined with the overall tumor response to bleomycin in this study, we believe that the cystic or vascular structures in the PMMTI form a pathway throughout the tumor, through which bleomycin may act and ultimately lead to a marked inflammatory and necrotic reaction in the tumor. The tumor developed a distinct border with the surrounding tissue in response to bleomycin. In the previous reports, most of the surgical cases failed to establish a negative incision, or a negative incision was not established (2). Excessive resection may have devastating consequences on the limbs and may eventually lead to limb disability. Therefore, the obvious inflammatory and necrotic reaction of the PMMTI after bleomycin injection played a guiding role in the complete surgical resection of the tumor.

Similar to previous PMMTI cases, we also observed a distinct myxoid background in the pathology of this patient, combined with the previously mentioned cystic and vascular structures within the tumor (2).

PMMTI is usually neither encapsulated nor clearly demarcated from normal tissue, which poses a challenge for its complete resection. The administration of bleomycin or similar chemotherapeutic drugs may cause the boundary of the tumor to become clear, therefore increasing the possibility of complete surgical resection. However, most chemotherapy and radiotherapy regimens in the existing cases have failed to yield satisfactory results in cases where the tumor cannot be completely removed (8, 9). Therefore, the consequences of bleomycin injection are unclear. Further studies are needed to verify whether a locally high concentration of bleomycin can directly cause tumor necrosis and bring benefits to the patient.

The potential mechanisms for the treatment of PMMTI with bleomycin have not yet been determined. We can only speculate on the mechanism of action of the drug based on the structural characteristics of PMMTI and the therapeutic effects of the drug itself. For other solid tumors with structures similar to that of PMMTI and tumors with the vasculature or myxoid stroma, injections with chemotherapy drugs, such as bleomycin, may prove effective when complete resection is difficult or impossible and when chemotherapy and radiotherapy have difficulty in achieving therapeutic effects. Such treatments could cause a similar inflammatory and necrotic response to the tumor as that observed in the present study and may benefit the patient or create a border between the tumor and surrounding tissue to guide complete resection. However, these effects need to be verified in future studies.

PMMTI is a rare disease; therefore, very few clinical treatment options exist. According to previous reports, complete resection can result in the best prognosis, but not all patients are eligible for complete resection. For patients who can undergo complete resection, reducing the damage to normal tissue is an important area to explore. In our opinion, combined with the structural characteristics of PMMTI, injection therapy is a worthwhile treatment option. Especially for children whose tumors cannot be completely removed, injection therapy may create better treatment conditions compared to those existing at present; however, the potential of injection therapy needs further clinical investigation.

PMMTI has only recently been identified, and the follow-up period is relatively short for most patients. Data on short-term mortality may not accurately reflect the prognosis of the disease. Some parents may have a low willingness to treat malignant tumors in infancy, especially in disadvantaged areas. In addition, some patients who lost to follow-up may have given up treatment.

Our study was limited by the inclusion of only one case. At the initial diagnosis, bleomycin was injected, because the tumor was considered to be lymphangioma. The choice of this treatment is accidental, and we do not have more clinical data to verify the therapeutic effect of bleomycin on PMMTI. The mechanism by which the drug works has yet not been fully elucidated. Although the pathology after resection was tested by immunohistochemistry, we could not determine whether this was a typical BCOR-ITD case because of the limitations of laboratory conditions and lack of further genetic testing. In addition, the follow-up time of patients was short, and long-term prognosis still needs to be followed.

In conclusion, we report the case of a 38-day-old patient with PMMTI who was treated with a bleomycin injection. To the best of our knowledge, this is the first study to directly use intratumoral injection of bleomycin or a similar chemotherapeutic drug to treat PMMTI. After injection of bleomycin, the tumor showed a marked inflammatory and necrotic response, resulting in a clear demarcation between the tumor and surrounding tissue. Resection was performed during the second hospitalization. 3 months after excision, no local recurrence was observed on re-examination.

We believe that injection therapy with chemotherapy drugs, such as bleomycin, may be a new breakthrough for patients with PMMTI who exhibit unclear demarcation between the tumor and surrounding tissue, which makes it difficult to perform complete resection. Being a rare disease, we have very limited clinical experience in treating such cases, and we hope that other clinicians may also consider the proposed treatment plan when treating similar cases.

## Data availability statement

The original contributions presented in the study are included in the article/supplementary material, further inquiries can be directed to the corresponding authors.

## Ethics statement

The studies involving human participants were reviewed and approved by the Ethics Committee of the Children's Hospital of Zhejiang University School of Medicine(2022-IRB-188). Written informed consent to participate in this study was provided by the participants' legal guardian/next of kin. Written informed consent was obtained from the minor(s)' legal guardian/next of kin for the publication of any potentially identifiable images or data included in this article.

## Author contributions

BY, QC, and YQ analyzed the data and wrote the manuscript. YZ, JW, AD, YC, WG, XH, and ZG contributed to clinical and instrumental data. All authors contributed to the article and approved the submitted version.

## Funding

This study was supported by the Natural Science Foundation of Zhejiang Province (Grant No. LY19H190005).

## Conflict of interest

The authors declare that the research was conducted in the absence of any commercial or financial relationships that could be construed as a potential conflict of interest.

## Publisher's note

All claims expressed in this article are solely those of the authors and do not necessarily represent those of their affiliated organizations, or those of the publisher, the editors and the reviewers. Any product that may be evaluated in this article, or claim that may be made by its manufacturer, is not guaranteed or endorsed by the publisher.
